# Complementary regional heterogeneity information from COPD patients obtained using oxygen-enhanced MRI and chest CT

**DOI:** 10.1371/journal.pone.0203273

**Published:** 2018-08-30

**Authors:** Yoshinori Fuseya, Shigeo Muro, Susumu Sato, Naoya Tanabe, Atsuyasu Sato, Kazuya Tanimura, Koichi Hasegawa, Kiyoshi Uemasu, Takeshi Kubo, Aki Kido, Koji Fujimoto, Yasutaka Fushimi, Hiroshi Kusahara, Naotaka Sakashita, Yoshiharu Ohno, Kaori Togashi, Michiaki Mishima, Toyohiro Hirai

**Affiliations:** 1 Department of Respiratory Medicine, Graduate School of Medicine, Kyoto University, Kyoto, Japan; 2 Department of Diagnostic Imaging and Nuclear Medicine, Graduate School of Medicine, Kyoto University, Kyoto, Japan; 3 Toshiba Medical Systems Corporation, Otawara, Tochigi, Japan; 4 Division of Functional and Diagnostic Imaging Research, Department of Radiology, Kobe University Graduate School of Medicine, Kobe, Japan; 5 Advanced Biomedical Imaging Research Center, Kobe University Graduate School of Medicine, Kobe, Japan; Central Michigan University College of Medicine, UNITED STATES

## Abstract

**Background:**

The heterogeneous distribution of emphysema is a key feature of chronic obstructive pulmonary disease (COPD) patients that typically is evaluated using high-resolution chest computed tomography (HRCT). Oxygen-enhanced pulmonary magnetic resonance imaging (OEMRI) is a new method to obtain information regarding regional ventilation, diffusion, and perfusion in the lung without radiation exposure. We aimed to compare OEMRI with HRCT for the assessment of heterogeneity in COPD patients.

**Methods:**

Forty patients with stable COPD underwent quantitative HRCT, OEMRI, and pulmonary function tests, including arterial blood gas analysis. OEMRI was also performed on nine healthy control subjects. We measured the severity of emphysema (percent low attenuation volume; LAV%) in whole lungs and the standard deviations (SDs) of the LAV% values of 10 isovolumetric partitions (SD-LAV) as an index of cranial-caudal heterogeneity. Similarly, relative enhancement ratios of oxygen (RERs) in whole lungs from OEMRI and SD-RER were analyzed.

**Results:**

COPD patients showed a lower mean RER than control subjects (12.6% vs 22.0%, p<0.01). The regional heterogeneity of the RERs was not always consistent with the LAV distribution. Both the HRCT (LAV% and SD-LAV) and the OEMRI (RER and SD-RER) indices were significantly associated with the diffusion capacity (DL_CO_) and partial pressure of oxygen in arterial blood (PaO_2_). The PaO_2_ was associated only with the heterogeneity index of HRCT (SD-LAV) (R^2^ = 0.39); however, the PaO_2_ was associated with both the mean RER and heterogeneity (SD-RER) in the multivariate analysis (R^2^ = 0.38).

**Conclusions:**

OEMRI-derived parameters were directly associated with oxygen uptake in COPD patients. Although the OEMRI-derived parameters were not identical to the HRCT-derived parameters, the cranial-caudal heterogeneity in HRCT or OEMRI was complementary to that in evaluations of oxygen uptake in the lungs. Functional imaging seems to provide new insights into COPD pathophysiology without radiation exposure.

## Introduction

Chronic obstructive pulmonary disease (COPD) is characterized by airflow limitation as a result of parenchymal destruction and airway disease. Parenchymal destruction is pathologically determined as pulmonary emphysema that includes loss of the alveolar surface area and the vascular bed[1], which may cause decreased diffusion capacity[2] and hypoxemia[3,4]. Pulmonary emphysema may be distributed heterogeneously, and this distribution pattern is a key feature of COPD patients.

High-resolution computed tomography (HRCT) is a standard technique used to quantify the severity of emphysema and its spatial distribution[5–9]. However, quantifying the spatial distribution heterogeneity can be challenging[8–10]. We previously reported that a more homogeneous cranial-caudal distribution of emphysema contributed to an accelerated progression of airflow limitation independent of the baseline whole-lung emphysema severity determined using HRCT[11]. Martinez et al. reported that lower lung-dominant emphysema predicted mortality in the medical therapy arm group of the National Emphysema Treatment Trial[12]. Thus, evaluating the whole-lung emphysema severity as well as the distribution of emphysematous changes is important.

However, quantitative CT analyses only assess regional morphological alterations in the lung and may not precisely reflect regional functional changes. Although significant associations exist between the emphysema severity and the diffusing capacity of the respiratory system (DL_CO_)[8], the correlation between respiratory functions and the emphysema severity on CT images is often poor[13]. Ventilation-perfusion mismatch that cannot be assessed by HRCT may play an important role in this discrepancy. Therefore, new functional imaging techniques that can determine spatial respiratory dysfunction are required.

Oxygen-enhanced pulmonary MRI (OEMRI) is a new method to obtain the functional index of the lung using inhaled oxygen as a contrast agent[14]. This method has advantages for the assessment of a diseased lung compared to pulmonary function tests and chest CT analysis, because it can evaluate regional oxygen transfer across the alveolus without radiation exposure. Recently, promising results have been reported by an investigation of the correlations of the OEMRI index and pulmonary function tests among patients with various lung diseases[15]; however, these OEMRI studies have evaluated whole-lung function, and no information is available concerning the heterogeneous distribution of diseased lesions in the lung in CT images. Therefore, we hypothesized that in addition to whole-lung image analysis, the cranial-caudal heterogeneity of the OEMRI index would provide further information on the relationship between regional lung parenchymal destruction and lung function. Specifically, we hypothesized that OEMRI might have advantages over HRCT for evaluating oxygen-transfer function capacity and arterial blood oxygenation in the emphysematous lung.

## Materials and methods

### Subjects

Forty patients with stable COPD were recruited from the outpatient clinic at Kyoto University Hospital between December 2012 and March 2014. The patients underwent CT scans, pulmonary function tests, including arterial blood gas analysis, and OEMRI. Nine healthy subjects were also enrolled in the present study as controls.

The detailed inclusion and exclusion criteria for the COPD patients are described in the online material (see online supplemental materials). The healthy subjects exhibited normal spirometry and had no current smoking habits. The ethics committee at Kyoto University approved the study (approval No. C664), and all patients and healthy subjects provided written informed consent for participation in the study.

### Pulmonary function tests

Pulmonary function tests were performed after inhalation of short-acting bronchodilators (400 μg of salbutamol and 80 μg of ipratropium)[11]. Spirometry, subdivisions of the lung volume, and DL_CO_ were measured using a Chestac-65V (Chest MI Corp, Tokyo, Japan). All procedures were conducted following the standard procedures of the ATS/ERS guidelines[16]. Arterial blood gas analysis was performed after resting in room air for 15 min in a sitting position using the RAPIDlab 1265 blood gas analyzer (Siemens Healthcare Diagnostics Inc., PA, USA).

### Quantitative HRCT

High-resolution CT scans at a slice thickness of 0.5 mm (Aquilion 64; Toshiba Medical Systems Co., Otawara, Japan) were acquired as previously reported[11,17,18]. A single CT scanner was used to avoid inter-scanner variability. Patients held their breath at full inspiration during acquisition of the chest CT scans. The CT images were acquired with 0.5-mm collimation, a scan time of 500 ms, a 120-kV peak, and automatic exposure control. The reconstruction algorithm was a lung algorithm (FC56) with automatic correction of the beam hardening effect. In addition to routine calibration using an air and water phantom, the CT values were adjusted using the tracheal air density[17].

### OEMRI

OEMRI was obtained with inhaled 100% oxygen as a T1 contrast agent during resting ventilation with a pneumatic belt to detect the respiratory phase. T1-weighted images (TI = 900 ms) were continually collected with a respiratory synchronized single shot FASE [Fast Asymmetric Spin Echo, sequentially reordered half-Fourier reconstructed short TE long echo train length FSE; TR, 5,500–11,000 ms depending on the respiratory cycle, TEeff, 8 ms, echo train length 65, and a 128x256 matrix with a filling factor of 50.8% (128x256 reconstruction matrix)] using a 1.5 T scanner (EXCELART Vantage; Toshiba Medical Systems Co., Otawara, Japan).

To obtain a single coronal section, subjects inhaled room air for 5 min, followed by 100% oxygen (15 L/min) using a non-rebreathing ventilation mask for 5 min and then room air again for 5 min. We obtained three coronal sections at three different locations: the center of the lung, including the carina and bilateral main bronchus, and 30 mm anterior and posterior to that section (see S1 Fig). The scan time for the MRI sequence was 5 min, and the total examination time was approximately 30 min. These procedures were similar to previously described methods[19,20].

### Image analysis of chest HRCT

The percentages of the LAV in the entire lung (LAV%) were analyzed semi-automatically according to a modified method using custom-made software as described elsewhere[11,21]. The cut-off level for LAV was defined as <-960 HU. The cranial-caudal heterogeneity of emphysema was assessed by calculating the SD of the LAV% values in 10 partitions with equal volumes (SD-LAV) as previously described[11,22]. Briefly, the lungs were divided into 12 equal volumes from the top to the bottom. The top and bottom parts were excluded because of the partial volume effect. The LAV was measured in each partition, and the LAV% in each partition was calculated by dividing the value by the partition volumes. Then, the SD of the LAV% values in the 10 partitions was calculated to obtain the SD-LAV. A higher SD-LAV represents a more heterogeneous distribution, and a lower SD-LAV represents a homogeneous distribution of emphysema[11].

### Image analysis of OEMRI

The relative enhancement ratio (RER) is the percentage change of the signal intensity in each pixel between the enhanced and baseline conditions and is an index for oxygen uptake.[23] The RER is calculated as follows:

$$\mathrm{RER}=\frac{\left|\mathrm{SIenhanced}-\mathrm{SIbaseline}\right|}{\left|\mathrm{SIbaseline}\right|}\times 100$$

The mean relative enhancement ratio (MRER) is the average of the RERs measured from regions of interest drawn to visually extract the lung parenchyma over both lungs on the coronal section by the author (Y. F.). Similar to the SD-LAV analysis, the SD-RER is calculated as a standard deviation of the MRER in 10 partitions of the overall MRER data (see S2 Fig). Higher and lower SD-RERs represent more heterogeneous and homogeneous distributions of the diseased lung, respectively (see S3 Fig).

### Statistical analysis

All statistical analyses were performed using the JMP 10 software (SAS Institute, Cary, NC, USA). Differences between the two groups were evaluated with Student’s t test or the Mann-Whitney U test when the data did not pass the normality test (Shapiro-Wilk test). Pearson’s bivariate correlation was used to determine the association between two variables, and Spearman's test was used for non-parametric data. To reduce a risk for multiple comparison bias, we focused on three representative parameters for lung function, such as FEV1, DL_CO_, and PaO_2_. A multivariate regression analysis was performed to evaluate the relative contributions of variables. p-values <0.05 were considered significant.

## Results

**Fig 1:**
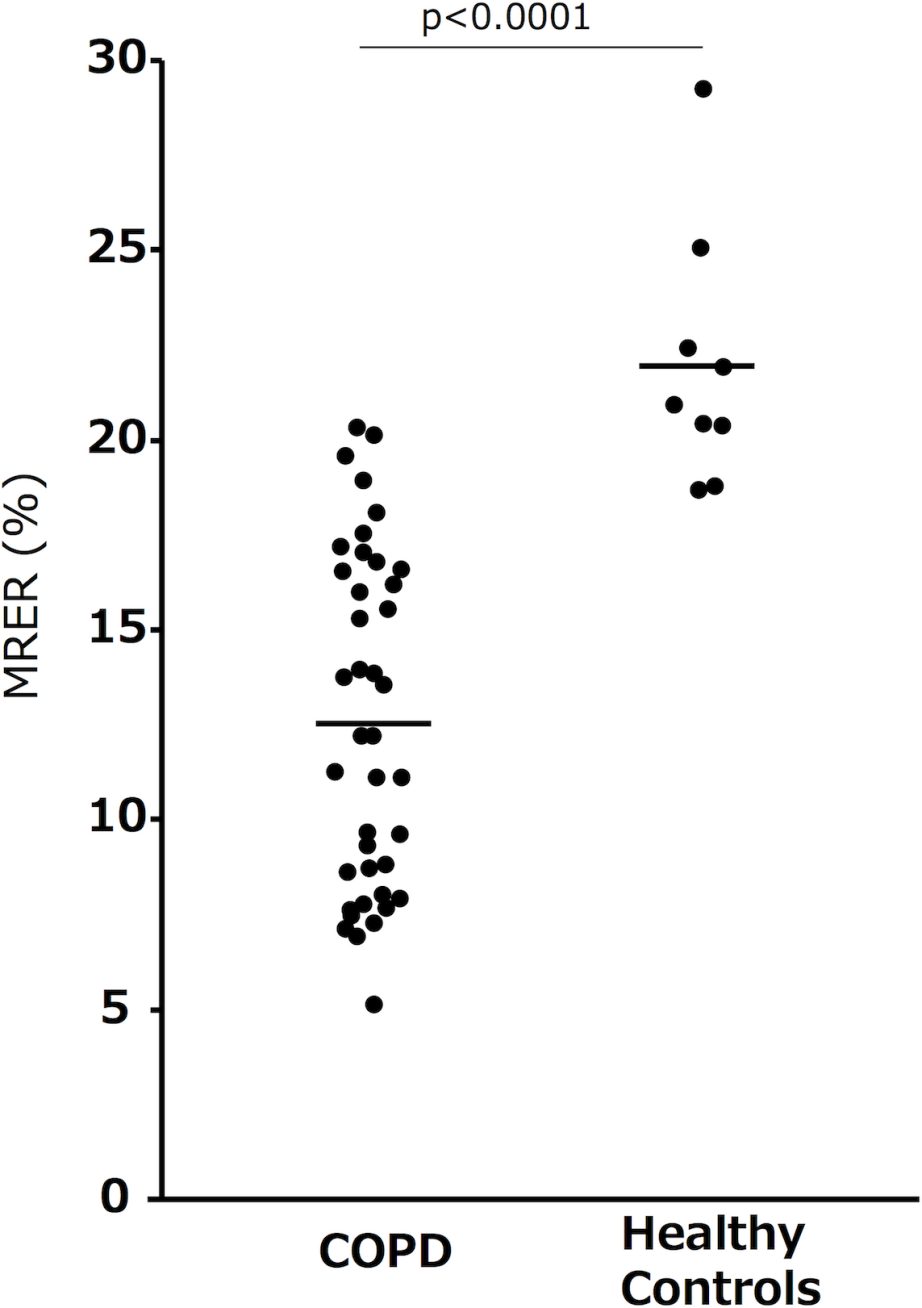
The MRERs of the COPD patients and healthy volunteers. The data are presented as medians (bar).

The characteristics of the COPD patients and control subjects are shown in Table 1. The COPD patients were older and had poorer lung function with the exception of %VC than the control subjects. The COPD patients showed a significantly lower MRER than the healthy volunteers (Fig 1, 12.6±4.4% vs 22.0±3.4%, p<0.0001). Representative RER maps are shown in S1 Fig.

**Table 1:** Characteristics of the study subjects.

Characteristics	COPD patients (N = 40)		Healthy controls (N = 9)			p-value
**Age**	70.5	(65.0, 76.8)	34.0		(31.5, 36.5)	<0.0001
**Height, m**	1.67	(1.62, 1.71)	1.70		(1.66, 1.76)	0.051
**Weight, kg**	61.5	(55.3, 67.0)	59.0		(58.4, 66.5)	0.53
**BMI, kg/m^2^**	21.5	(20.3, 23.7)	20.9		(19.9, 23.5)	0.71
**Smoking status,** **current:former:never**	13:27:0		0:2:7			
**Smoking history,** **pack-years**	50.0	(41.3, 72.5)	7.0		(4.0, 10.0)	0.018
**FEV_1_, L**	1.80	(1.19, 2.19)	3.91		(3.79, 4.06)	<0.0001
**%FEV_1_, %**	64.8	(45.3, 73.7)	96.9		(95.4, 105.6)	<0.0001
**FEV_1_/FVC, %**	54.9	(40.9, 63.3)	88.7		(78.7, 92.4)	<0.0001
**GOLD classification,** **1/2/3/4**	7/19/11/3		-			
**VC, L**	3.56	(3.01, 3.83)	4.57		(4.13, 4.86)	0.0002
**%VC, %**	98.6	(89.1, 109.2)	97.9		(89.8, 107.5)	0.87
**RV/TLC, %**	39.3	(34.3, 45.3)	27.6		(23.0, 36.7)	0.0016
**DL_CO_, mL/min/mmHg**	12.2	(8.8, 17.6)	31.1		(27.0, 32.9)	<0.0001
**%DL_CO_, %**	53.1	(37.6, 73.7)	101.2		(90.9, 110.3)	<0.0001
**pH**	7.42	(7.41, 7.45)	-		-	-
**PaO_2_, Torr**	76.3	(72.6, 85.1)	-		-	-
**PaCO_2_, Torr**	39.5	(37.2, 41.9)	-		-	-

The data are presented as medians (25th, 75th percentiles) unless otherwise indicated.

BMI, body mass index; FEV_1_, forced expiratory volume in 1 second; %FEV_1_, percentage of FEV_1_ predicted; FVC, forced vital capacity; VC, vital capacity; RV, residual volume; TLC, total lung capacity; D_LCO_, diffusing capacity to alveolar ventilation; PaO_2_, partial pressure of oxygen in arterial blood; PaCO_2_, partial pressure of arterial carbon dioxide.

**Fig 2:**
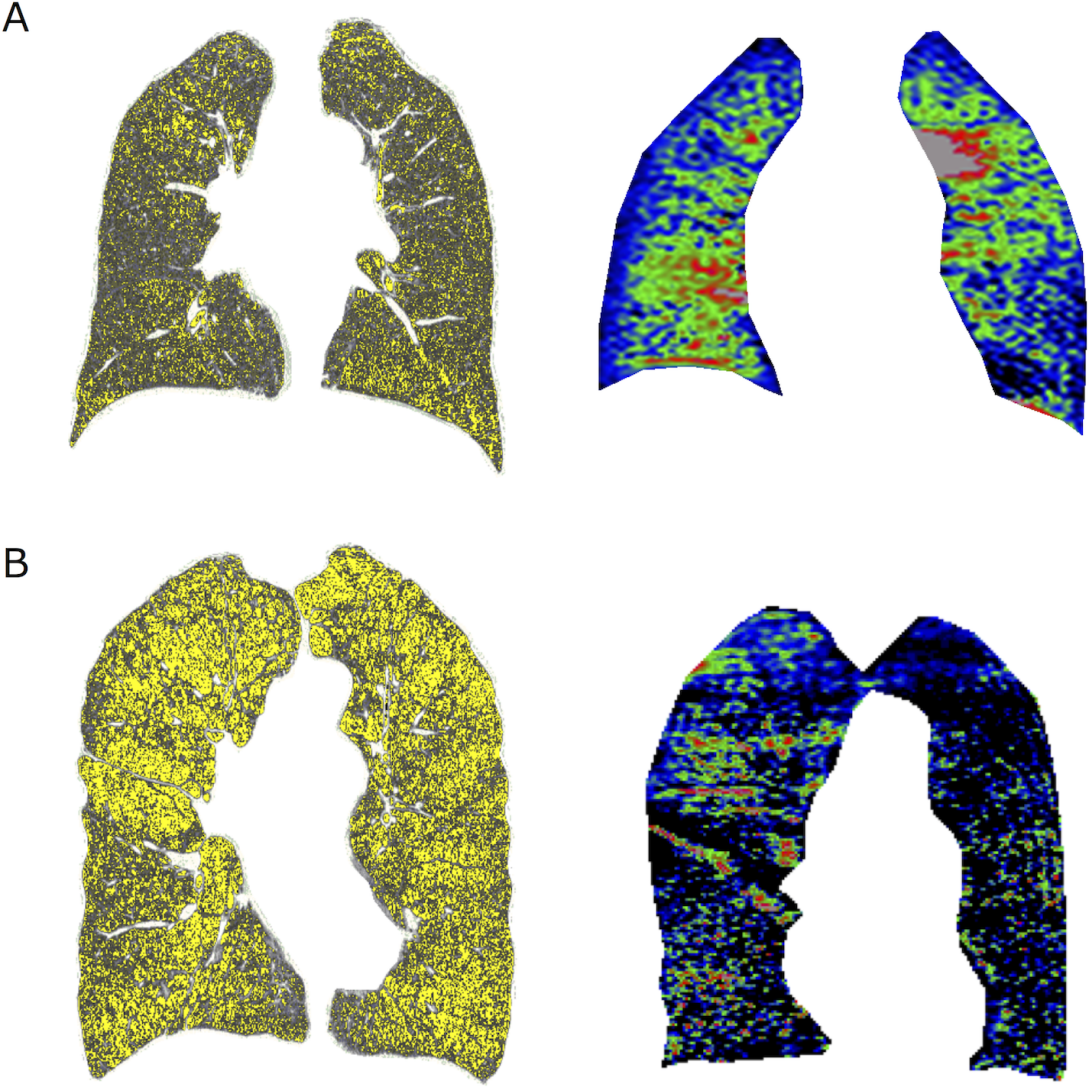
Examples of CT and OEMRI coronal images from COPD patients. LAV (~-960 HU) is indicated in yellow in the CT image. **A.** In this case, the coronal CT showed strong emphysematous changes in the left lower lung field, and the OEMRI also showed low oxygen uptake in the left lower lung. Both the coronal CT and OEMRI indicated similar results. **B.** In this severe emphysematous case, the coronal CT showed the same degree of emphysematous changes in both upper lung fields, although the OEMRI showed lower oxygen uptake in the left upper lung field. The low RER regions in the OEMRI did not necessarily match the low attenuation regions in the CT images.

Fig 2 shows a comparison of representative HRCT and OEMRI coronal images from the COPD patients. In the case shown in Fig 2A, the coronal CT showed strong emphysematous changes in the left lower lung field, and the OEMRI also showed low oxygen uptake in the left lower lung. Both the coronal CT and OEMRI images indicated similar results. In another case shown in Fig 2B, the coronal CT showed the same degree of emphysematous changes in both upper lung fields, although the OEMRI showed lower oxygen uptake in the left upper lung field.

**Fig 3:**
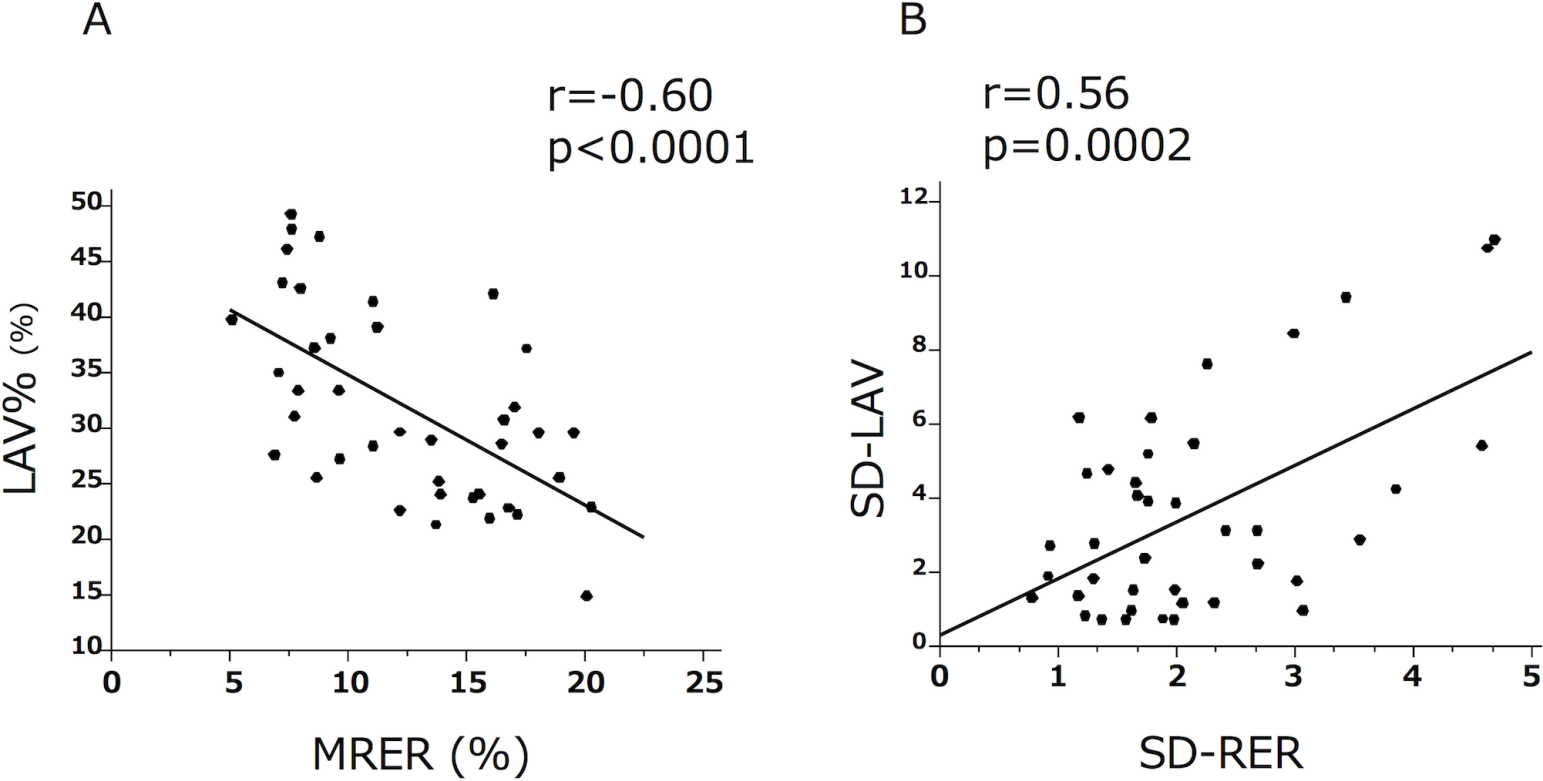
The relationship between quantitative CT and OEMRI. **A.** The MRER showed a significant correlation with the LAV%. **B.** The SD-RER and SD-LAV were significantly correlated in the COPD patients.

Regarding the whole-lung evaluation, the MRER showed a significant but moderate correlation with the LAV% (Fig 3A, r = -060, p<0.0001) in the COPD patients. The heterogeneity indices (SD-RER and SD-LAV) were also significantly but moderately correlated in the COPD patients (Fig 3B, r = -0.56, p = 0.0002). These results suggested that the LAV% and MRER as well as the SD-LAV and SD-RER were not identical.

Bivariate and multivariate analyses between the LAV% or SD-LAV and the pulmonary function tests, including the arterial blood analysis, are shown in Tables 2 and 3. The whole-lung emphysema severity index (LAV%) was significantly associated with the forced expiratory volume in 1 second (FEV_1_) (r = -0.58, p<0.0001), DL_CO_ (r = -0.68, p<0.0001), and the partial pressure of oxygen in arterial blood (PaO_2_) (r = -0.34, p = 0.03). The cranial-caudal emphysema heterogeneity index (SD-LAV) was significantly associated with the DL_CO_ (r = -0.53, p = 0.0004) and PaO_2_ (r = -0.64, p<0.0001) but not the FEV_1_. The multivariate analysis showed that the LAV% but not the SD-LAV was associated with the FEV_1_, whereas both the LAV% and SD-LAV were significant factors for estimation of the DL_CO_. Interestingly, the SD-LAV was the only association factor for the PaO_2_ estimation.

**Table 2:** Bivariate analyses between the CT and pulmonary function test indices.

	LAV%		SD-LAV	
	r	p-value	r	p-value
**FEV_1_**	-0.60	<0.0001	-0.20	0.2
**DL_CO_**	-0.64	<0.0001	-0.44	0.004
**PaO_2_**	-0.39	0.01	-0.62	<0.0001

Pearson’s bivariate correlation

**Table 3:** Stepwise multivariate regression analysis showing the relative contribution of each CT variable to the prediction of the pulmonary function test results.

	FEV_1_			DL_CO_			PaO_2_		
Variables	β	p-value	R^2^	β	p-value	R^2^	β	p-value	R^2^
**LAV%**	-0.49	0.0004		-0.46	0.001			0.2	
**SD-LAV**		0.9		-0.25	0.046		-0.62	<0.001	
**Age**	-0.34	0.01		-0.26	0.04			0.4	
**BMI**		0.2			0.2			0.4	
**Cumulative R^2^**			0.46			0.52			0.39

Age, BMI, LAV%, and SD-LAV were included as candidate independent variables.

FEV_1_, forced expiratory volume in 1 second; DL_CO_, diffusing capacity to alveolar ventilation

PaO_2_, partial pressure of oxygen in arterial blood; LAV, low attenuation volume; SD, standard deviation; BMI, body mass index

Bivariate and multivariate analyses of the MRER or SD-RER and the pulmonary function tests are shown in Tables 4 and 5. The whole-lung oxygenation index (MRER) was significantly associated with the FEV_1_ (r = 0.35, p<0.03), DL_CO_ (r = 0.59, p<0.0001), and PaO_2_ (r = 0.52, p = 0.0006). In contrast to the MRER, the SD-RER was not significantly associated with any pulmonary function test. However, the multivariate analysis demonstrated that the SD-RER was associated with the PaO_2_ independent of the MRER. The MRER but not the SD-RER was significantly associated with the DL_CO_.

**Table 4:** Bivariate analyses of the oxygen-enhanced MRI and pulmonary function test indices.

	MRER		SD-RER	
	r	p-value	r	p-value
**FEV_1_**	0.30	0.06	-0.14	0.4
**DL_CO_**	0.58	<0.0001	-0.067	0.7
**PaO_2_**	0.54	0.0003	-0.28	0.08

Pearson’s bivariate correlation

**Table 5:** Stepwise multivariate regression analysis showing the relative contribution of each variable of the oxygen-enhanced MRI for the prediction of the pulmonary function test results.

	FEV_1_			DL_CO_			PaO_2_		
Variables	β	p-value	R^2^	β	p-value	R^2^	β	p-value	R^2^
**MRER**		0.1		0.58	<0.0001		0.55	0.0001	
**SD-RER**		0.9			0.8		-0.30	0.03	
**Age**	-0.37	0.01		-0.43	0.0006			0.4	
**BMI**	0.40	0.005			0.07			0.5	
**Cumulative R^2^**			0.39			0.53			0.38

Age, BMI, MRER, and SD-RER were included as candidate independent variables.

FEV_1_, forced expiratory volume in 1 second; DL_CO_, diffusing capacity to alveolar ventilation

PaO_2_, partial pressure of oxygen in arterial blood; MRER, mean relative enhancement ratio; RER, relative enhancement ratio; SD, standard deviation; BMI, body mass index

## Discussion

In the present study, we found that the cranial-caudal emphysema heterogeneity detected by both HRCT and OEMRI had a significant influence on the overall lung diffusion capacity (DL_CO_) and PaO_2_. We also found that the cranial-caudal heterogeneity of the oxygen-transfer function (SD-RER) provided additional information about the PaO_2_ independent of the whole-lung oxygen-transfer function index assessed by OEMRI (MRER).

In addition, we found that the cranial-caudal emphysema heterogeneity detected by HRCT (SD-LAV) but not the LAV% was associated with the PaO_2_ in the multivariate analysis. Although studies have shown the influence of the heterogeneous distribution of emphysema on lung function by CT[6,11,22], the present study is the first to successfully identify a significant association between whole-lung pulmonary function, including PaO_2_, and cranial-caudal heterogeneity by comparing indices derived from both OEMRI and HRCT.

COPD is the leading cause of respiratory failure. Oxygenation is a critical matter for COPD patients, and hypoxemia is associated with a poor quality of life and prognosis[4,24–26]. In this study, we assessed arterial blood gases (PaO_2_) and elucidated a strong association with an oxygen-transfer function derived from a new functional imaging technique (OEMRI).

Our colleague previously showed that the whole-lung averaged RER (MRER) of COPD patients exhibited a good correlation with indices obtained from quantitative CT analysis[20]. Similarly, we showed a significant correlation (Fig 2A) and found that COPD patients had a significantly lower MRER than control subjects (Fig 1). We also found a strong association between the MRER and important indices related to O_2_ transfer, such as DL_CO_ and PaO_2_, in COPD patients (Tables 4 and 5). However, the MRER was not associated with the severity of airflow limitation (r = 0.30, p = 0.06) in COPD patients. These findings suggest that OEMRI has an advantage in the assessment of lung gas transfer impairment as a result of parenchymal destruction and ventilation-perfusion mismatch rather than airflow limitation, which is a consequence of airway lesions and parenchymal destruction.

Although our control subjects were not age matched with the COPD patients, normal elderly subjects with normal oxygenation may have similar or better distributions than COPD patients.

Numerous studies have shown that the extent of the emphysematous lesion in whole lungs is important for assessment of COPD in clinical practice[2,3,5–8,11,18,21,27, 28]. For example, we reported that emphysematous changes assessed by a CT scan predicted respiratory mortality in COPD patients[28]. Because CT can simultaneously measure the spatial distribution of emphysema, recent reports have also focused on the heterogeneous distribution of emphysema[10,11,13]. We reported that upper lung-dominant emphysema was associated with a specific genetic background[29] and that a cranial-caudal homogeneous distribution of emphysema contributed to further longitudinal changes in the FEV_1_[11]. In this cross-sectional study, we found that the SD-LAV had additional effects on the DL_CO_ estimation and was significantly associated with the PaO_2_. Therefore, we argue that a highly heterogeneous distribution of structural destruction may have an important negative impact on oxygenation in the respiratory system, probably due to severe ventilation-perfusion mismatch. In fact, the SD-RER showed a weak but significant association with the SD-LAV (Fig 2B).

Interestingly, the CT-derived parameters (the LAV% and SD-LAV) were associated with the DL_CO_ in the multivariate analysis, whereas the SD-LAV but not the LAV% was associated with the PaO_2_ (Table 3). Since the COPDGene results showed that emphysema severity on a quantitative chest CT scan did not predict hypoxemia[30], we naturally assumed that LAV alone was not sufficient to explain gas transfer and arterial oxygenation. LAV simply reflects airspace enlargement and the morphologic alveolar destruction index but not perfusion. We speculate that the SD-LAV may somehow reflect the mismatch or uneven distribution of ventilation in diseased lungs.

In contrast, since both the DL_CO_ and PaO_2_ are strongly associated with ventilation, perfusion, and their ratios[31], the OEMRI-derived parameter may represent DL_CO_ and PaO_2_ with some cooperation. In a diseased lung, the regional distribution and heterogeneity of destruction may have complementary information for these functional data[32,33]. Therefore, SD-LAV may be associated with the DL_CO_ in addition to the LAV%. These speculations need to be further investigated; however, the present study suggests that evaluating both the overall emphysema severity and the distribution of emphysema is important for predictions of gas transfer and hypoxemia, whereas assessment using functional imaging may be a better investigative method for gas transfer and hypoxemia.

Considering the overall assessments and heterogeneity, different mechanisms could be applied to the OEMRI parameters compared to those obtained with HRCT. The overall average RER (the MRER) already represents much of the overall transfer function of oxygen in the lung[34], because OEMRI can measure tissue oxygenation. This possibility may be a reason for the lack of significant associations between the SD-RER and the DL_CO_ or PaO_2_ in the bivariate analyses, whereas the SD-LAV showed significant associations with these variables (Tables 2 and 4). However, because a high SD-RER indicated severe spatial heterogeneity (mismatch) of oxygenation, the multiple regression analysis showed that the SD-RER had a negative impact on the PaO_2_ (Table 5). This finding suggests that severe heterogeneity of emphysema diminishes overall oxygenation of the lungs.

Moreover, the RER may more accurately reflect O_2_ behavior than CO. This possibility could explain why the relationship between the SD-RER and the PaO_2_ or DL_CO_ was not identical and suggested that OEMRI had a distinct advantage in evaluating the lung capacity for blood oxygenation compared to pulmonary function tests or HRCT. Another possible mechanism underlying the differences between the HRCT and OEMRI indices may be small airway narrowing, which cannot be assessed using HRCT. OEMRI can detect the normal lung parenchyma in CT images where ventilation is poor due to small airway narrowing, because these areas have low oxygen uptake. These speculations should also be elucidated in a future study.

The present study has some limitations. First, the present study enrolled a smaller number of subjects than previous studies; however, we found similar and consistent results with our previous larger study using only HRCT[11]. This small size is an institutional limitation; however, we minimized the variation of machines and procedures during imaging and successfully obtained similar results. Therefore, this issue may not be a true limitation. The second problem is that OEMRI is time consuming. However, since OEMRI involves no radiation exposure, it is a potential alternative to a chest CT examination. Third, we could not use the same imaging method to evaluate cranial-caudal heterogeneity in HRCT and OEMRI. We obtained only 3 coronal sections from OEMRI, whereas the entire lung field was evaluated by HRCT. Moreover, we found a tendency for the MRER to be higher in the posterior section than in the anterior section, which might have resulted from a gravity effect. We are hopeful that this effect can be resolved in the future. Nevertheless, we successfully found similar results using both HRCT and OEMRI; moreover, OEMRI may have distinct advantages in estimating whole-lung oxygenation functions that reflect ventilation and perfusion without radiation exposure.

In the future, with development of higher resolution and increased accessibility, OEMRI will become available to assess regional functions of patients with severe breathlessness and/or hypoxemia. Then, this information will help in evaluation of patients with COPD and other chronic pulmonary diseases.

## Conclusions

OEMRI has the advantage of providing visual information regarding the regional transfer function of the lung. The heterogeneous distribution of emphysematous lesions assessed by HRCT and OEMRI can provide a deeper understanding of the pathophysiology of COPD patients.

## Supporting information

S1 Fig **Representative RER maps for each of the three locations from a healthy control subject (A) and a COPD patient (B).** The images from the COPD patient are dark compared with those from the healthy control.
(PDF)

S2 Fig Calculation of cranial-caudal heterogeneity of the relative enhancement ratio (SD-RER). The SD-RER was calculated as the standard deviation of the MRER in 10 parts of the overall MRER data (three sections).
(PDF)

S3 Fig Examples of heterogeneous and homogeneous emphysema. Although the MRERs shown in A and B were almost similar, the SD-RER was higher in A than in B.
(PDF)

## Abbreviations

BMI: body mass index
COPD: chronic obstructive pulmonary disease
CT: computed tomography
DL_CO_: diffusion capacity of carbon monoxide
FEV_1_: forced expiratory volume in one second
GOLD: Global Initiative for Chronic Obstructive Lung Disease
HU: Hounsfield unit
LAV: low attenuation volume
MRI: magnetic resonance imaging
OEMRI: oxygen-enhanced pulmonary magnetic resonance imaging
RER: relative enhancement ratio
MRER: mean relative enhancement ratio
RV: residual volume
SD: standard deviation
TLC: total lung capacity
PaO_2_: partial pressure of oxygen in arterial blood
PaCO_2_: partial pressure of carbon dioxide in arterial blood
