# Supplementary figures and images for: Complementary regional heterogeneity information from COPD patients obtained using oxygen-enhanced MRI and chest CT
Source: PLoS One. 2018 Aug 30;13(8):e0203273. doi: 10.1371/journal.pone.0203273 (PMC6117056; doi:10.1371/journal.pone.0203273)

# S1 Fig

A

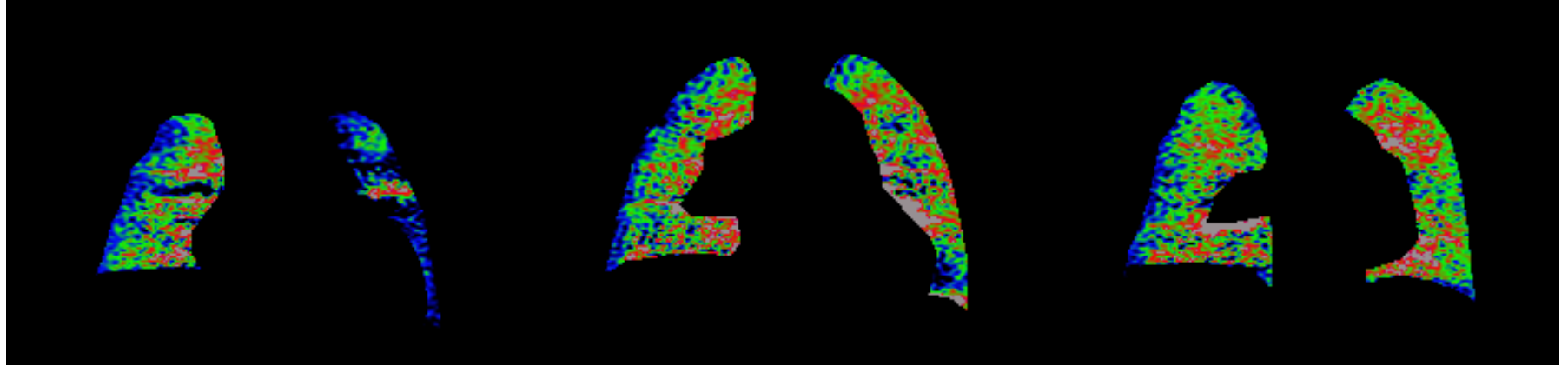

B

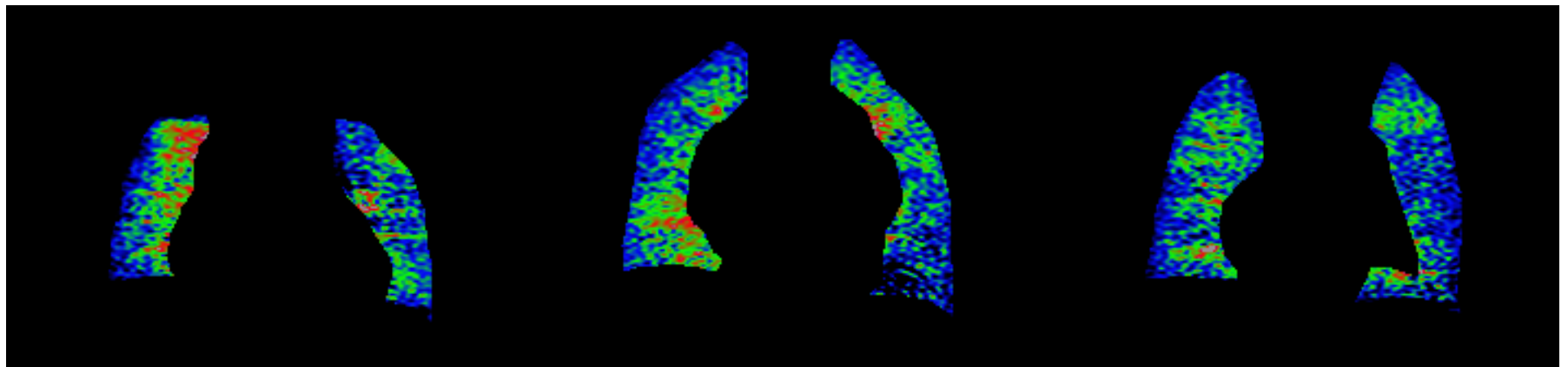

0%

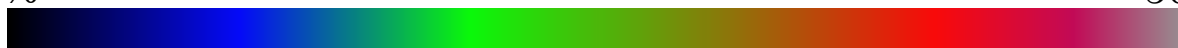

50%

Supplement: S1 Fig — Representative RER maps for each of the three locations from a healthy control subject (A) and a COPD patient (B). The images from the COPD patient are dark compared with those from the healthy control. (PDF) [file pone.0203273.s001.pdf]

S2 Fig

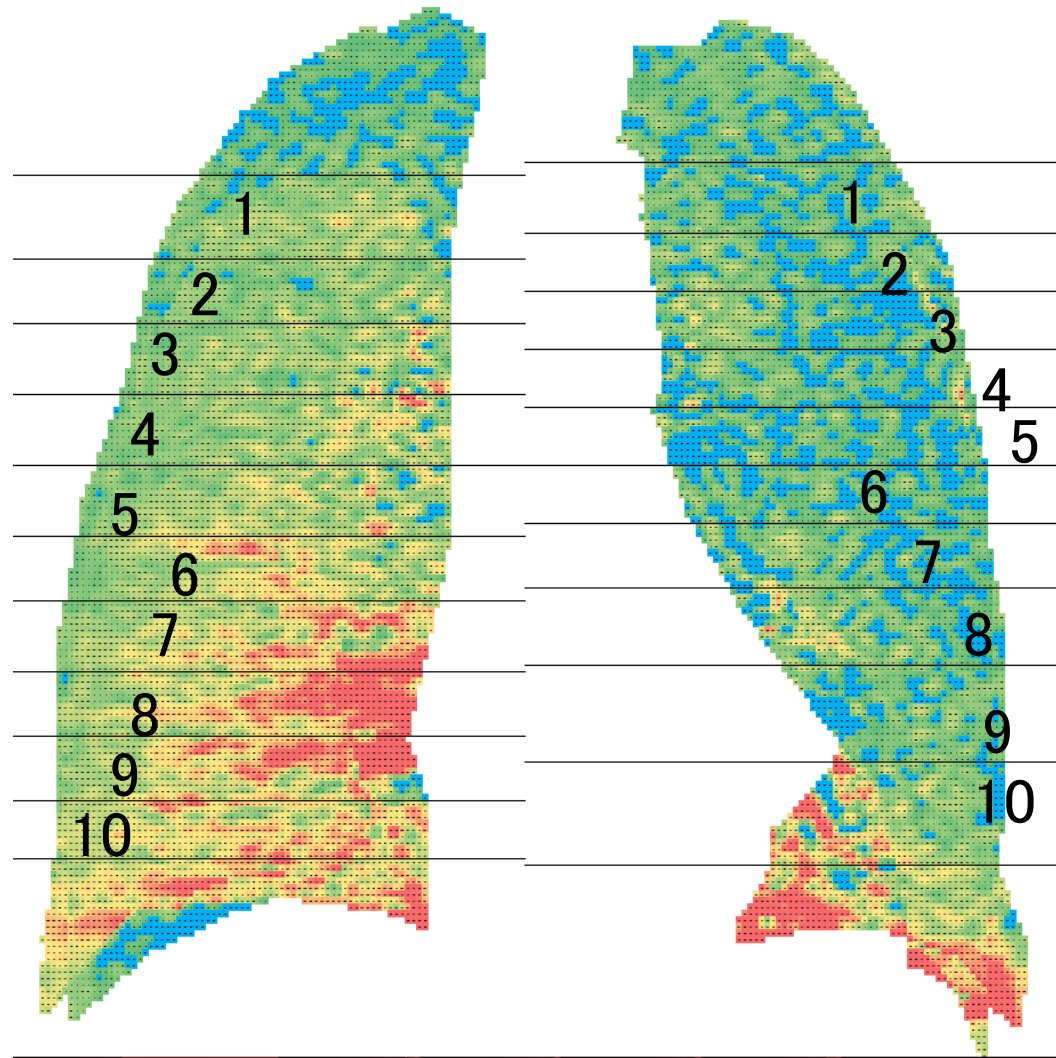

Supplement: S2 Fig — The SD-RER was calculated as the standard deviation of the MRER in 10 parts of the overall MRER data (three sections). (PDF) [file pone.0203273.s002.pdf]

S3 Fig

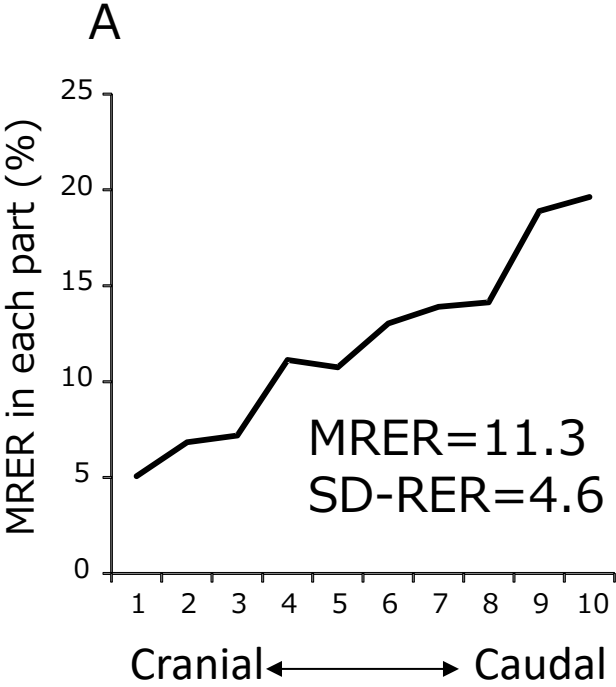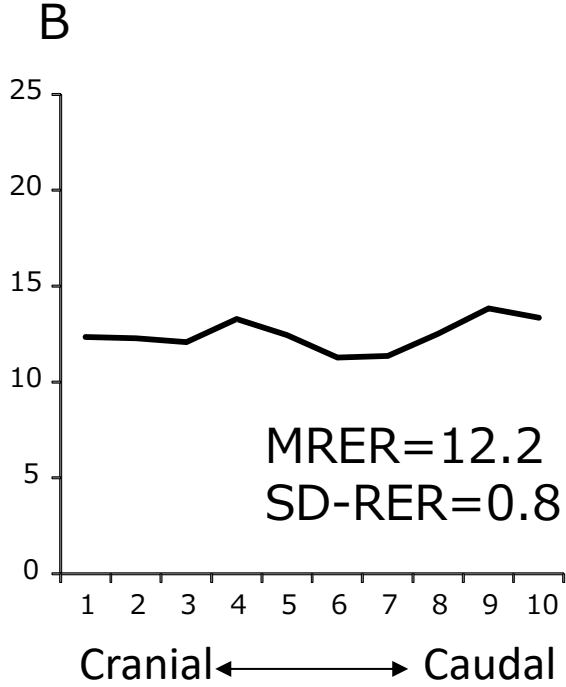

Supplement: S3 Fig — Although the MRERs shown in A and B were almost similar, the SD-RER was higher in A than in B. (PDF) [file pone.0203273.s003.pdf]
